# Corrigendum: Demystifying Brain Tumor Segmentation Networks: Interpretability and Uncertainty Analysis

**DOI:** 10.3389/fncom.2021.651959

**Published:** 2021-01-29

**Authors:** Parth Natekar, Avinash Kori, Ganapathy Krishnamurthi

**Affiliations:** Department of Engineering Design, Indian Institute of Technology Madras, Chennai, India

**Keywords:** interpretability, CNN, brain tumor, segmentation, uncertainty, activation maps, features, explainability

In the original article, we neglected to include the funder **Robert Bosch Center for Data Science and Artificial Intelligence (RBCDSAI)**, **CR1920ED617RBCX008562**.

We would like to add the above grant information to the Acknowledgment section as follows:

“This work was funded by the Robert Bosch Center for Data Science and Artificial Intelligence (RBCDSAI), under project number CR1920ED617RBCX008562 (Interpretability for Deep Learning Models in Healthcare).”

The authors apologize for this error and state that this does not change the scientific conclusions of the article in any way. The original article has been updated.

