# Supplementary figures and images for: Demystifying Brain Tumor Segmentation Networks: Interpretability and Uncertainty Analysis
Source: Front Comput Neurosci. 2020 Feb 7;14:6. doi: 10.3389/fncom.2020.00006 (PMC7025464; doi:10.3389/fncom.2020.00006)

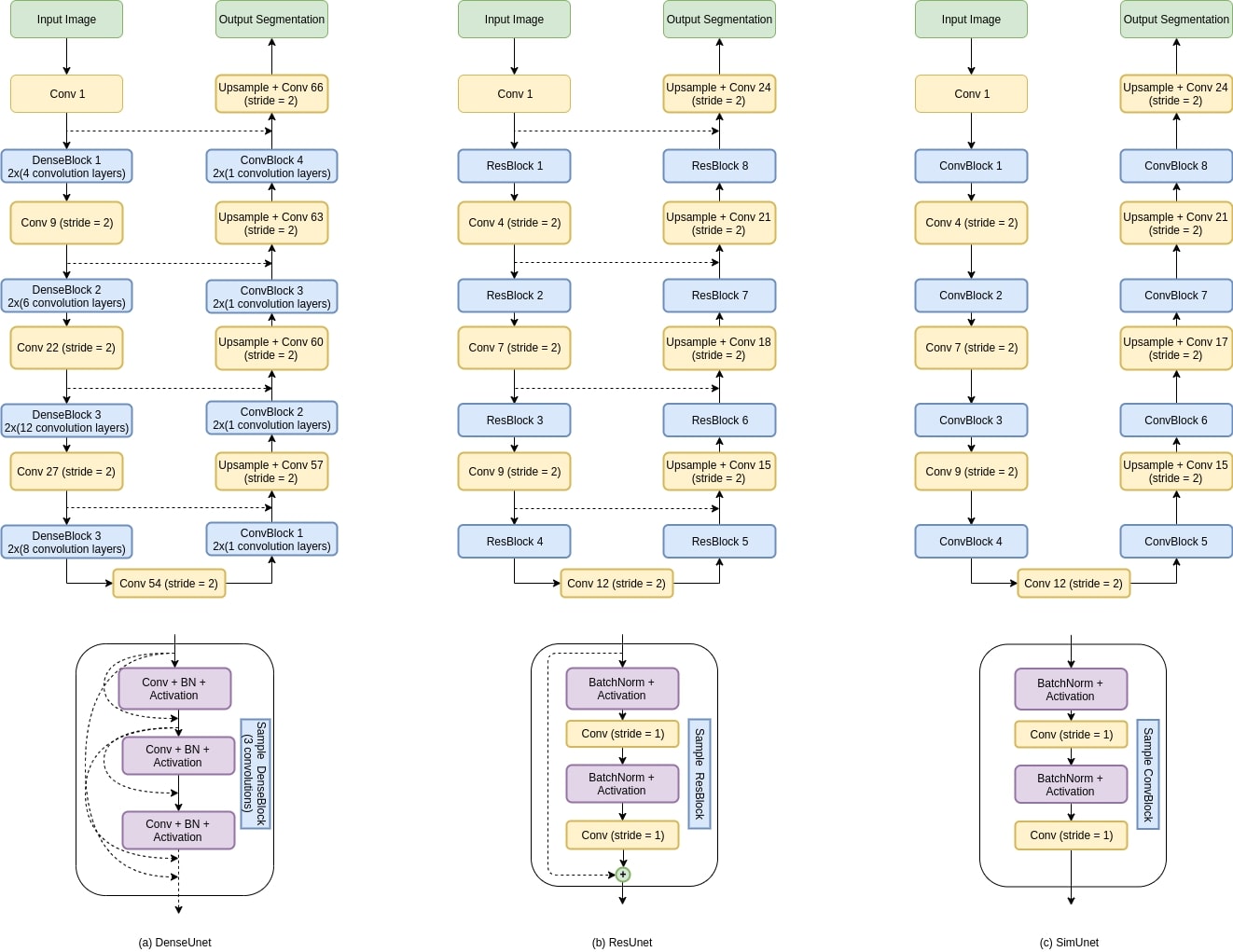

Supplement: Supplementary Figure 1 — Network Architectures used in our study. [file Image_1.jpg]

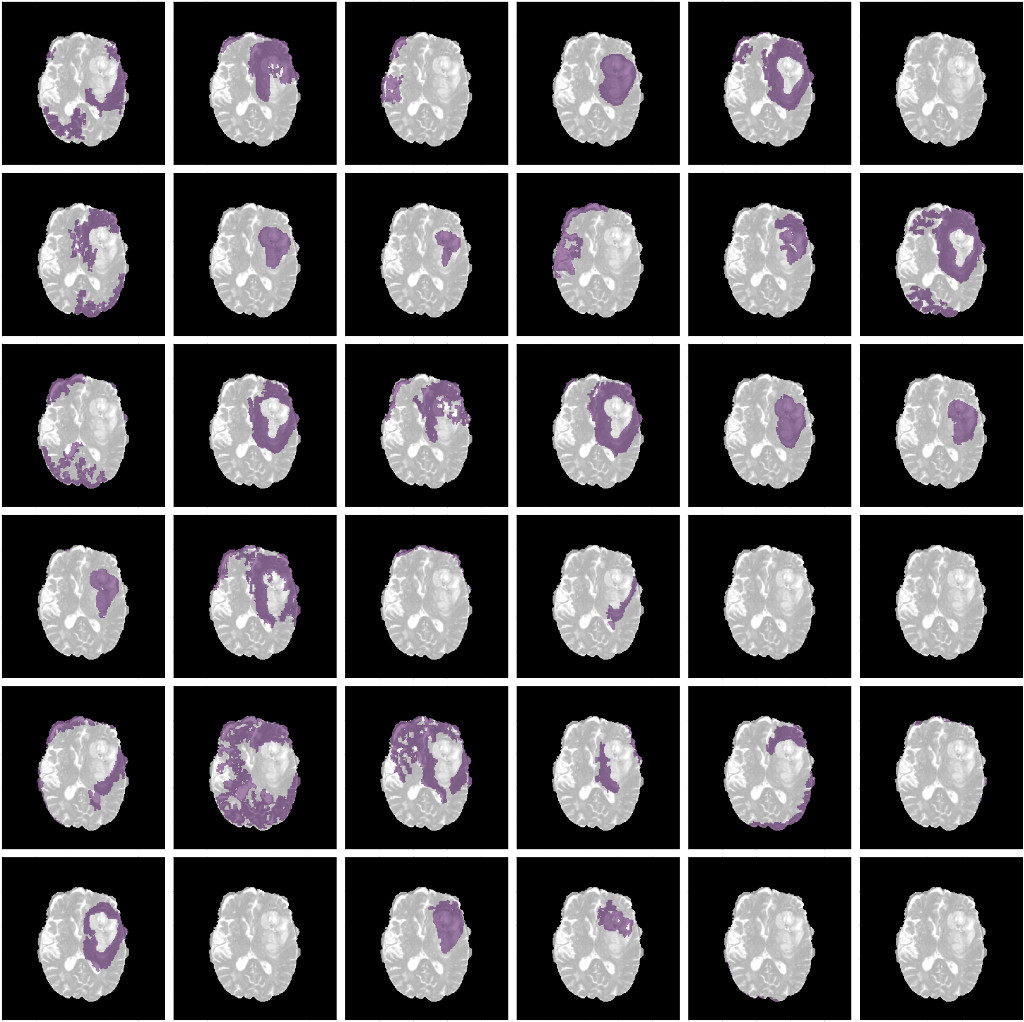

Supplement: Supplementary Figure 2 — Concepts learned by filters of a particular layer of the ResUnet for an input image (Conv Layer 21). [file Image_2.jpg]

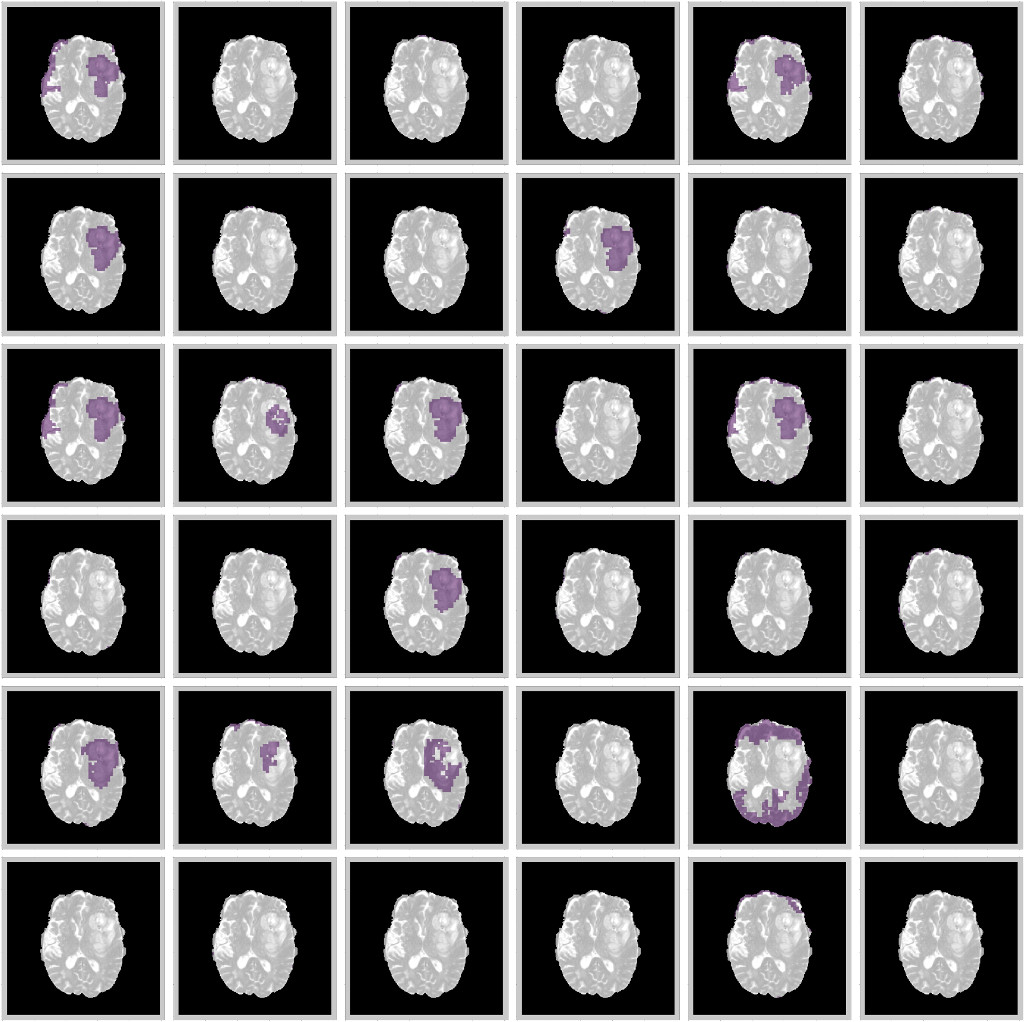

Supplement: Supplementary Figure 3 — Concepts learned by filters of a particular layer of the DenseUnet for an input image (Encoding Block 1, Conv 2). [file Image_3.jpg]

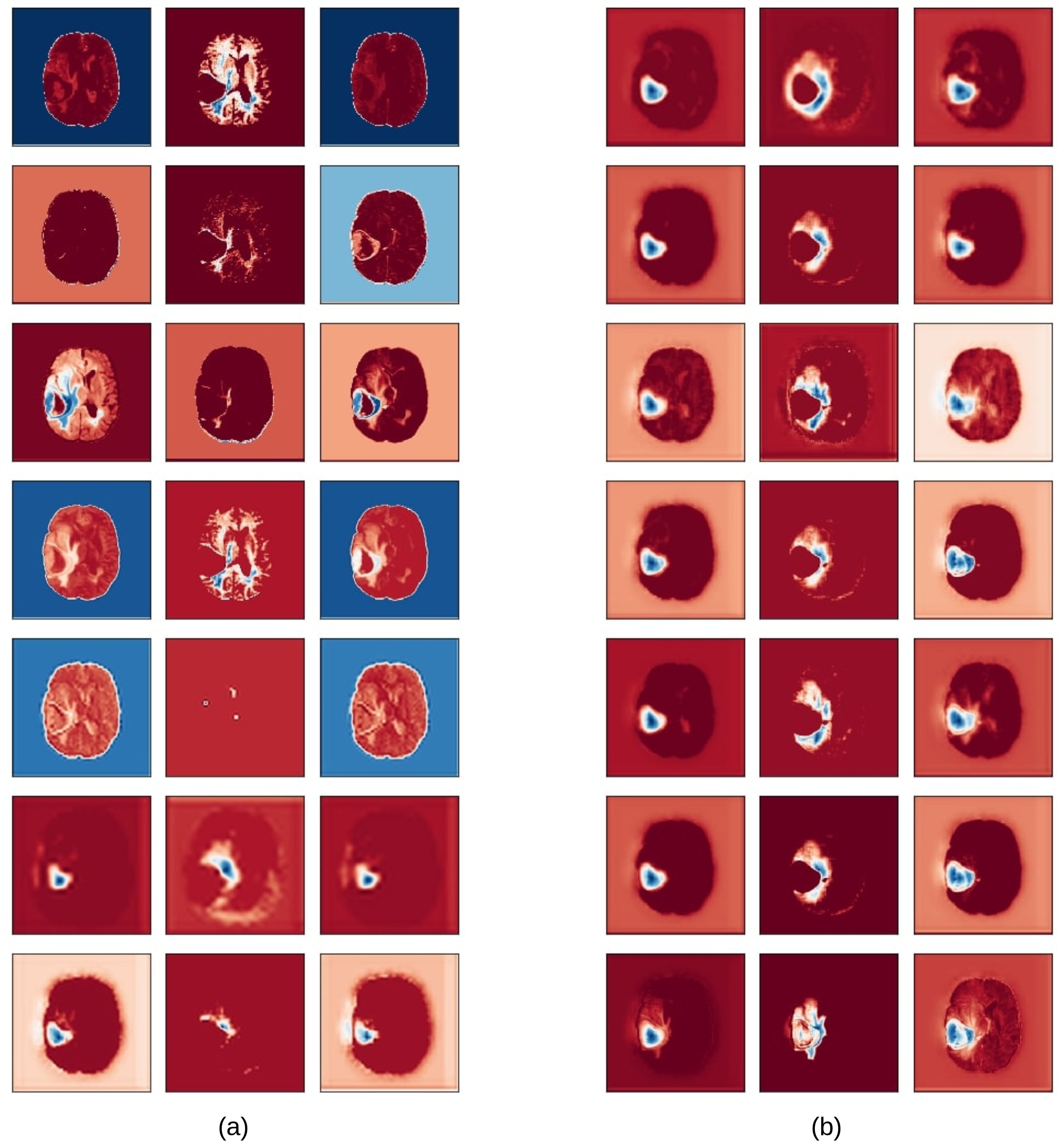

Supplement: Supplementary Figure 4 — Grad-CAM results for consecutive layers of the ResUnet [view: top to bottom, column (A), followed by top to bottom, column (B)]. [file Image_4.png]

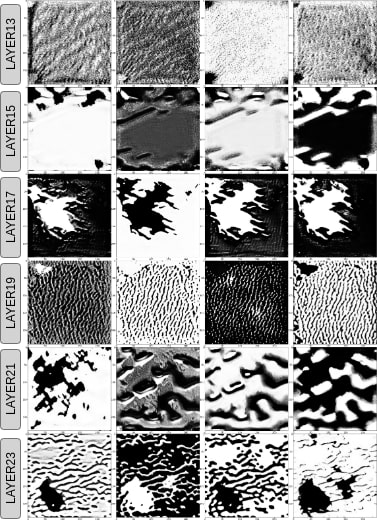

Supplement: Supplementary Figure 5 — Activation maps for layers of the ResUnet. [file Image_5.jpg]

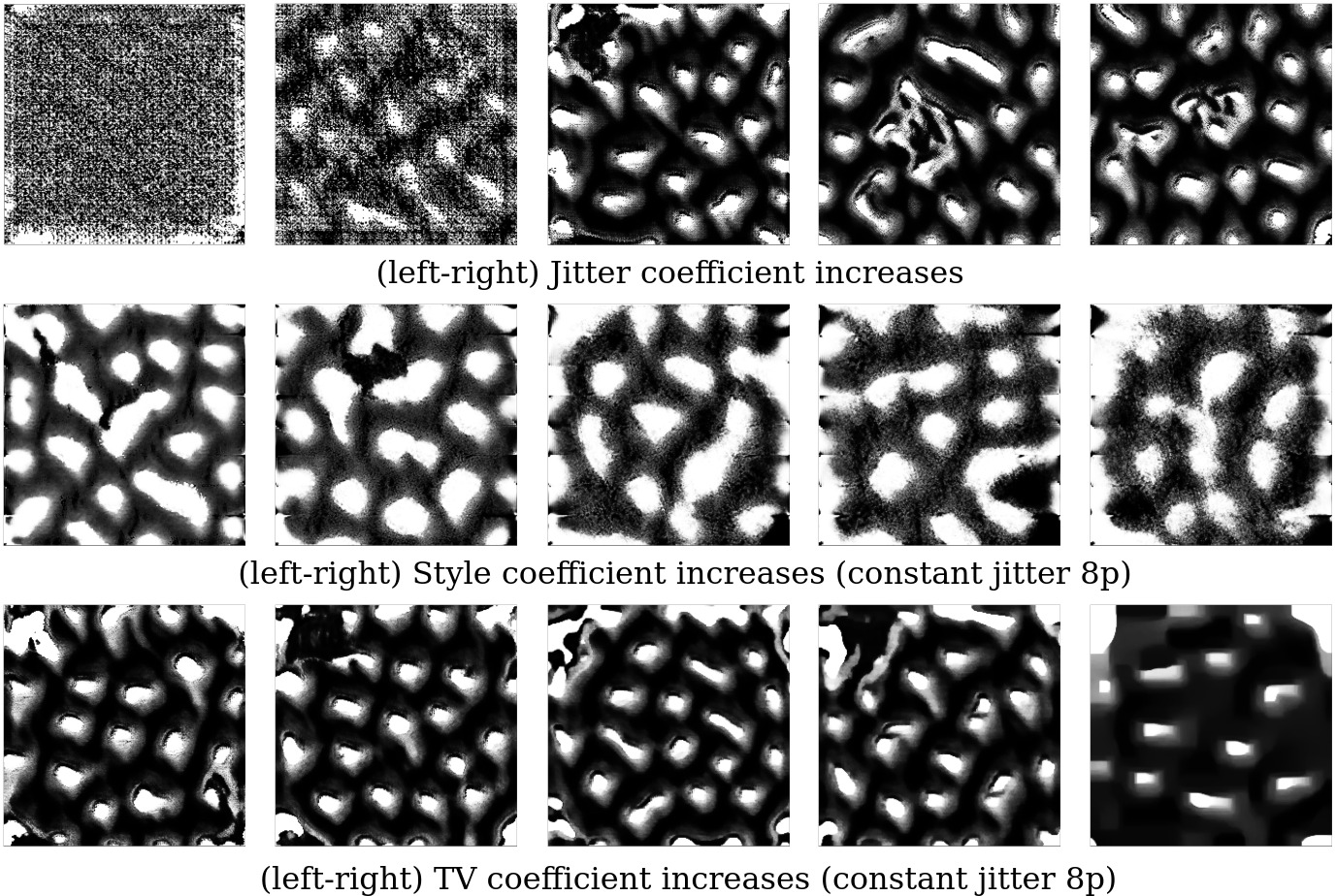

Supplement: Supplementary Figure 6 — Effect of independently changing hyperparamaters for each regularizer. (Top) Jitter coefficient increases [0 pixels, 1p, 6p, 12p, 20p]. (Middle) Style Coefficient increases [10−2, 10−1, 1, 5, 10]. (Bottom) Total Variation regularization increases [10−7, 10−6, 10−5, 10−4, 10−3] to smoothen image. [file Image_6.jpg]

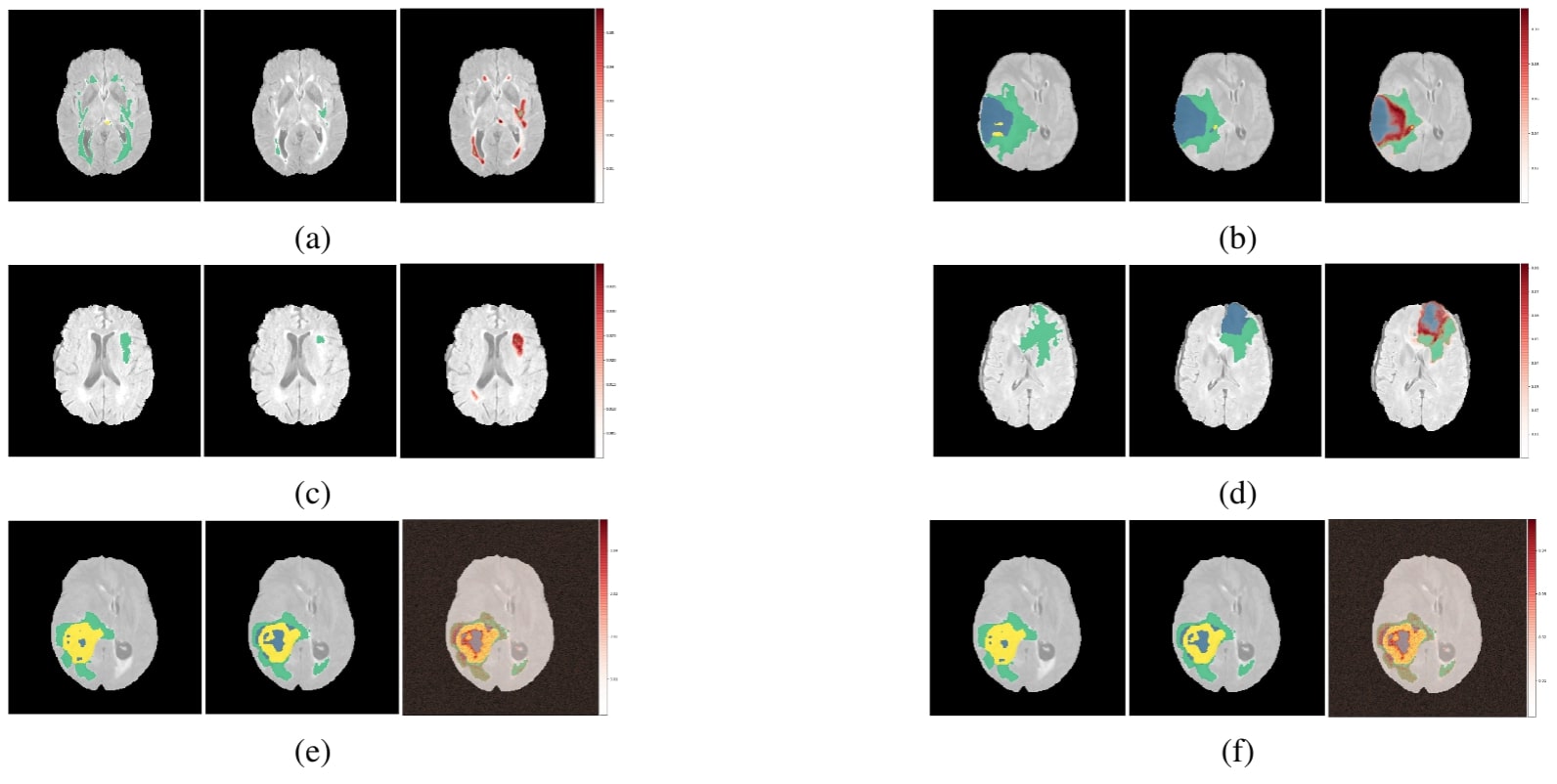

Supplement: Supplementary Figure 7 — Uncertainty estimations (shown in red) for the DenseUnet (a–d) and ResUnet (e,f). Ground Truth (Left), Model Prediction (Middle), and Uncertainty (Right). [file Image_7.jpg]
